# Risk‐adjusted safety analysis of the oral JAK2/IRAK1 inhibitor pacritinib in patients with myelofibrosis

**DOI:** 10.1002/jha2.591

**Published:** 2022-10-20

**Authors:** Naveen Pemmaraju, Claire Harrison, Vikas Gupta, Srdan Verstovsek, Bart Scott, Stephen T. Oh, Francesca Palandri, Haifa Kathrin Al‐Ali, Marta Sobas, Mary Frances McMullin, Ruben Mesa, Sarah Buckley, Karisse Roman‐Torres, Alessandro Vannucchi, Abdulraheem Yacoub

**Affiliations:** ^1^ The University of Texas, MD Anderson Cancer Center Houston Texas USA; ^2^ Guy's and St Thomas’ NHS Foundation Trust London UK; ^3^ Princess Margaret Cancer Centre University Health Network Toronto Ontario Canada; ^4^ Fred Hutchinson Cancer Research Center Seattle Washington USA; ^5^ Washington University School of Medicine St Louis Missouri USA; ^6^ IRCCS Azienda Ospedaliero‐Universitaria di Bologna Istituto di Ematologia “Seràgnoli” Bologna Italy; ^7^ Krukenberg Cancer Center Halle University Hospital Halle Halle Germany; ^8^ Department of Hematology, Blood Neoplasms and Bone Marrow Transplantation Wroclaw Medical University Wrocław Poland; ^9^ Queen's University Belfast Belfast UK; ^10^ UT Health San Antonio MD Anderson Cancer Center San Antonio Texas USA; ^11^ CTI BioPharma Seattle Washington USA; ^12^ University of Florence, Azienda Ospedaliera Universitaria Careggi Florence Italy; ^13^ The University of Kansas Cancer Center Westwood Kansas USA

**Keywords:** myelofibrosis, myeloproliferative neoplasms, pacritinib, safety

## Abstract

The safety profile of the novel oral JAK2/IRAK1 inhibitor pacritinib in patients with cytopenic myelofibrosis was described in the Phase 2 PAC203 and Phase 3 PERSIST‐2 studies. To account for longer treatment durations on the pacritinib arms compared to best available therapy (BAT), we present a risk‐adjusted safety analysis of event rates accounting for different time on treatment. While the rate of overall events was higher on pacritinib compared to BAT, the rate of fatal events was lower, and there was no excess in bleeding, cardiac events, secondary malignancy, or thrombosis on pacritinib, including in patients with severe thrombocytopenia.

1

Pacritinib is an oral JAK2/IRAK1 inhibitor that was recently approved in the United States in February 2022 for patients with myelofibrosis (MF) who have a platelet count below 50 × 10^9^/L. This approval marks pacritinib as the third‐in‐class approved JAK inhibitor for patients with intermediate/higher risk MF, after ruxolitinib and fedratinib, and the only JAK inhibitor recommended for patients with severe thrombocytopenia,[1] which is prevalent in approximately 35% of the MF population.[2] Approval was based on data from the randomized Phase 3 PERSIST‐2 study (2014–2016), with additional supportive data from the second‐line Phase 2 PAC203 study (2017–2019) [3] in patients with advanced MF. [4] Cross‐over on the best available treatment (BAT) arm of PERSIST‐2 confounded the safety analysis of this study, which compared pacritinib to BAT, including the JAK1/2 inhibitor ruxolitinib. Patients randomized to BAT were able to cross over to pacritinib at 24 weeks or earlier in the setting of disease progression, and 51% (50/98) of patients on BAT did so (including 22 of 44 [50%] patients receiving ruxolitinib as BAT). Thus, while adverse events (AEs) were reported for the entire study duration in patients randomized to pacritinib, AEs were only reported on the BAT arm for the initial treatment period, resulting in an imbalance between arms in time at risk for treatment‐emergent AEs.

As patients with MF, particularly those with severe cytopenias, are at risk for multiple disease complications, including infection, bleeding, thrombotic, and cardiovascular events, **[** **5** **–** **9** **]** it is important to consider how new therapies will impact these risks. Recently, JAK inhibitors have come under increased scrutiny due to specific, emerging toxicities seen with drugs in this class, with the United States Food and Drug Administration (FDA) now requiring product label warnings regarding the increased risk of serious cardiac events, thromboses, cancer, deaths, and infections for all JAK inhibitors, including the JAK1/3 inhibitor tofacitinib and the JAK1/2 inhibitor baricitinib, agents approved currently in rheumatoid arthritis.[10, 11] An increased risk of herpes zoster reactivation has specifically been reported for the JAK1/2 inhibitor ruxolitinib. [12]

To comprehensively describe the safety profile of pacritinib, we present a risk‐adjusted analysis of these safety risks for pacritinib compared to BAT, including ruxolitinib, accounting for differential time on treatment. This analysis focuses on pacritinib 200 mg BID, as this is the FDA approved dose for treatment of MF.

Patients with MF treated with pacritinib 200 mg BID on PERSIST‐2 and PAC203 were included, as were patients randomized to BAT on PERSIST‐2, in a pooled analysis. The following AE types were analyzed: cardiac events (by Standardized MedDRA Query [SMQ]), heart failure events (determined by medical review), major adverse cardiac events (MACE, defined as fatal cardiac events or any ischemic stroke or myocardial infarction), thrombotic events (venous, arterial, and embolic, including myocardial infarction and ischemic stroke), bleeding events (by SMQ), infection events (by System Order Class, as well as subcategories of infection determined by medical review), and secondary malignancies (including nonmelanoma skin cancer, determined by medical review). Risk‐adjusted incidence rates were reported per 100 patient‐years and were calculated as 100 × (number of patients with an event)/(total patient‐years of drug exposure until the event for patients with an event, otherwise until the end of drug exposure).

A total of 160 patients were treated with pacritinib 200 mg BID (*n* = 106 from PERSIST‐2 and 54 from PAC203), and 98 patients were treated with BAT (including *n* = 44 with ruxolitinib). Baseline characteristics are shown in Table 1. The mean duration of therapy was longer on pacritinib (6.5 months on PERSIST‐2 and 6.0 months on PAC203) than BAT (4.9 months).

**TABLE 1:** Baseline patient and disease characteristics

	**PAC203**	**PERSIST‐2**			**Pooled Analysis**
**Characteristic**	**PAC 200 mg BID** *n*= 54	**PAC 200 mg BID** *n* = 106	**BAT** *n* = 98	**BAT = RUX** *n* = 44	**PAC 200 mg BID** *n* = 160
Age (years), median (range)	69 (37, 85)	67 (39, 85)	68 (32, 83)	68 (42, 83)	68 (37, 85)
Female gender, *n* (%)	22 (41%)	44 (42%)	45 (46%)	15 (34%)	66 (41%)
ECOG PS ≥2, *n* (%)	8 (15%)	12 (11%)	18 (18%)	10 (23%)	20 (13%)
PLT (×10^9^/L), median (IQR)^1^	59 (29, 91)	55 (36, 93)	57 (29, 81)	61 (35, 91)	57 (33, 93)
PLT < 50 × 10^9^/L, *n* (%)^1^	24 (44%)	47 (44%)	42 (43%)	17 (39%)	71(44%)
HB < 10 g/dl, *n* (%)	41 (76%)	62 (59%)	54 (55%)	23 (52%)	103 (64%)
Receives RBC transfusions, *n* (%)^2^	34 (63%)	49 (46%)	47 (48%)	19 (43%)	83 (52%)
Peripheral blasts ≥1%, *n* (%)	32 (59%)	48 (45%)	46 (47%)	27 (61%)	80 (50%)
Primary MF, *n* (%)	37 (69%)	82 (77%)	60 (61%)	22 (50%)	119 (74%)
DIPSS high risk, *n* (%)	14 (26%)	29 (27%)	26 (27%)	12 (27%)	43 (27%)
Prior JAKi exposure, *n* (%)	54 (100%)	51 (48%)	52 (53%)	32 (73%)	105 (66%)

Abbreviations: BAT, best available therapy; BID, twice daily; DIPSS, Dynamic International Prognostic Scoring System; ECOG PS, Eastern Cooperative Oncology Group Performance Status; HB, hemoglobin; IQR, interquartile range; JAKi, JAK inhibitor; MF, myelofibrosis; PAC, pacritinib; PLT, platelets; RBC, red blood cell; RUX, ruxolitinib.

^1^ Baseline platelet count not available for all patients in safety population.

^2^ At any point in the 90 days prior to first dose.

As shown in Table 2, the rate of all‐grade and grade ≥3 AEs was higher in the pooled pacritinib group compared to BAT, whereas the rate of fatal events was higher on BAT, including ruxolitinib, both overall and in patients with baseline platelet count <50 × 10^9^/L.

**TABLE 2:** Risk‐adjusted rates of treatment‐emergent adverse events (AEs) in all patients and in the subset of patients with baseline platelet count <50 × 10^9^/L. Data are presented as event rate per 100 patient‐years, followed by number of patients with events divided by total person‐years at risk for first event

	**PAC203**	**PERSIST‐2**			**Pooled Analysis**
**AE/100 pt‐yrs** (events/pt‐yrs)^1^	**PAC 200 mg BID *n* = 54**	**PAC 200 mg BID *n* = 106**	**BAT *n* = 98**	**BAT = RUX *n* = 44**	**PAC 200 mg BID *n* = 160**
**AE overview**					
Any event					
All pts	**2063** (54/2.6)	**1390** (100/7.2)	**903** (87/9.6)	**1468** (41/2.8)	**1570** (154/9.8)
PLT < 50 × 10^9^/L	**2609** (24/0.9)	**2171** (46/2.1)	**1064** (38/3.6)	**1408** (15/1.1)	**2303** (70/3.0)
Grade ≥3 event					
All pts	**252** (41/16.3)	**250** (76/30.4)	**167** (48/28.7)	**158** (20/12.7)	**250** (117/46.7)
PLT < 50 × 10^9^/L	**509** (23/4.5)	**371** (39/10.5)	**246** (26/10.6)	**188** (8/4.3)	**413** (62/15.0)
Fatal event					
All pts	**10** (3/29.6)	**12** (8/65.6)	**22** (9/41.5)	**27** (5/18.4)	**12** (11/95.2)
PLT < 50 × 10^9^/L	**20** (3/14.8)	**23** (6/25.8)	**48** (8/16.6)	**81** (5/6.2)	**22** (9/40.6)
**Cardiac events**					
Cardiac event^2^					
All pts	**101** (22/21.7)	**62** (34/55.3)	**81** (27/33.5)	**67** (10/14.8)	**73** (56/77.0)
PLT < 50 × 10^9^/L	**121** (13/10.8)	**77** (16/20.7)	**168** (19/11.3)	**230** (8/3.5)	**92** (29/31.5)
Cardiac grade ≥3					
All pts	**7** (2/28.2)	**11** (7/64.5)	**23** (9/39.8)	**11** (2/18.0)	**10** (9/92.7)
PLT < 50 × 10^9^/L	**15** (2/13.4)	**16** (4/24.7)	**52** (8/15.4)	**35** (2/5.7)	**16** (6/38.1)
MACE^3^					
All pts	**0** (0/29.6)	**0** (0/65.7)	**5** (2/41.4)	**5** (1/18.5)	**0** (0/95.3)
PLT < 50 × 10^9^/L	**0** (0/14.8)	**0** (0/25.8)	**12** (2/16.4)	**16** (1/6.3)	**0** (0/40.6)
Heart failure^4^					
All pts	**0** (0/29.6)	**0** (0/65.7)	**2** (1/41.4)	**0** (0/18.5)	**0** (0/95.3)
PLT < 50 × 10^9^/L	**0** (0/14.8)	**0** (0/25.8)	**6** (1/16.4)	**0** (0/6.3)	**0** (0/40.6)
QT prolongation					
All pts	**15** (4/27.0)	**3** (2/64.3)	**7** (3/40.6)	**0** (0/18.5)	**7** (6/91.4)
PLT < 50 × 10^9^/L	**0** (0/14.8)	**0** (0/25.8)	**6** (1/16.8)	**0** (0/6.3)	**0** (0/40.6)
**Bleeding events**					
Bleeding event^2^					
All pts	**105** (23/21.9)	**98** (45/45.8)	**129** (40/31.1)	**127** (18/14.1)	**100** (68/67.8)
PLT < 50 × 10^9^/L	**239** (18/7.5)	**133** (23/17.3)	**270** (26/9.6)	**229** (10/4.4)	**165** (41/24.8)
Bleeding grade ≥3					
All pts	**14** (4/28.9)	**29** (17/59.2)	**17** (7/40.8)	**17** (3/18.1)	**24** (21/88.0)
PLT < 50×10^9^/L	**21** (3/14.1)	**36** (8/22.3)	**31** (5/16.4)	**33** (2/6.1)	**30** (11/36.4)
**Malignancy events**					
Malignancy^5^					
All pts	**0** (0/29.6)	**8** (5/63.7)	**7** (3/40.8)	**11** (2/17.8)	**5** (5/93.3)
PLT < 50 × 10^9^/L	**0** (0/14.8)	**17** (4/24.2)	**6** (1/16.3)	**17** (1/5.8)	**10** (4/39.0)
Skin SCC/BCC^4^					
All pts	**0** (0/29.6)	**5** (3/64.2)	**7** (3/40.8)	**11** (2/17.8)	**3** (3/93.8)
PLT < 50 × 10^9^/L	**0** (0/14.8)	**8** (2/24.7)	**6** (1/16.3)	**17** (1/5.8)	**5** (2/39.5)
**Infection events**					
Infection event^6^					
All pts	**103 (23/22.3)**	**124** (51/41.2)	**88** (30/34.2)	**80** (12/15.1)	**116** (74/63.6)
PLT < 50 × 10^9^/L	**125** (13/10.4)	**188** (26/13.8)	**119** (15/12.6)	**113** (5/4.4)	**161** (39/24.2)
Viral infection^4^					
All pts	**7** (2/29.2)	**5** (3/65.1)	**12** (5/41.1)	**11** (2/18.3)	**5** (5/94.3)
PLT < 50 × 10^9^/L	**7** (1/14.8)	**8** (2/25.3)	**12** (2/16.5)	**0** (0/6.3)	**8** (3/40.1)
Zoster reactivation^7^					
All pts	**0** (0/29.6)	**0** (0/65.7)	**2** (1/41.5)	**6** (1/18.3)	**0** (0/95.3)
PLT < 50 × 10^9^/L	**0** (0/14.8)	**0** (0/25.8)	**0** (0/16.8)	**0** (0/6.3)	**0** (0/40.6)
Fungal infection^4^					
All pts	**10** (3/29.1)	**5** (3/64.1)	**12** (5/40.8)	**6** (1/18.3)	**6** (6/93.1)
PLT < 50 × 10^9^/L	**21** (3/14.3)	**8** (2/24.7)	**19** (3/16.2)	**0** (0/6.3)	**13** (5/39.0)
**Other event types**					
Encephalopathy^8^					
All pts	**0** (0/29.6)	**0** (0/65.7)	**0** (0/41.7)	**0** (0/18.5)	**0** (0/95.3)
PLT < 50 × 10^9^/L	**0** (0/14.8)	**0** (0/25.8)	**0** (0/16.8)	**0** (0/6.3)	**0** (0/40.6)
Thrombosis^9^					
All pts	**10** (3/29.4)	**2** (1/65.7)	**2** (1/41.0)	**6** (1/17.8)	**4** (4/95.1)
PLT < 50 × 10^9^/L	**7** (1/14.7)	**0** (0/25.8)	**6** (1/16.1)	**18** (1/5.5)	**3** (1/40.6)

Abbreviations: BAT, best available therapy; BCC, basal cell carcinoma; BID, twice daily; MACE, major adverse cardiac event; PAC, pacritinib; PLT, platelets; pt‐yrs, patient‐years; RUX, ruxolitinib; SCC, squamous cell carcinoma.

^1^ Events per 100 pt‐yrs are calculated as 100 times the number of patients with an event divided by the cumulative time on treatment for each patient until the first AE for patients with an event otherwise the last dose of treatment.

^2^ Defined by Standardized MedDRA Query (SMQ);.

^3^ Defined as any fatal cardiac event (by SMQ) or by ischemic stroke or myocardial infarction of any grade.

^4^ Determined by medical review.

^5^ All events within the systems order class “neoplasms benign, malignant, and unspecified” excluding acute leukemia, myelofibrosis, and benign tumors.

^6^ All events within the Systems Order Class “Infection.”

^7^ Any infection with the term “zoster” or “shingles.”

^8^ Any event with the term “encephalopathy” or “Wernicke's.”

^9^ Arterial thrombosis, venous thrombosis, thromboembolism, ischemic stroke, and type 1 myocardial infarction.

Cardiac events, including high‐grade events, occurred at slightly lower rates on pacritinib compared to BAT. QT prolongation events were more common on PAC203 compared to PERSIST‐2, likely due to increased electrocardiographic monitoring in the former. MACE was not reported in any pacritinib‐treated patients, whereas it was on BAT, although rates were low. Bleeding and thrombosis occurred at similar rates on pacritinib and BAT, including in patients with baseline platelet count <50 × 10^9^/L. Rates of thrombosis were highest in patients treated with ruxolitinib. Malignant neoplasms, including nonmelanoma skin cancer, occurred at similar rates on pacritinib and BAT. Rates of these events were highest in ruxolitinib‐treated patients. Infection occurred slightly more frequently on pacritinib compared to BAT, although there was no increase in risk of herpes zoster reactivation or fungal infection noted. There were no cases of encephalopathy reported.

These data, which account for differing times at risk for AEs, show that the safety profile of pacritinib 200 mg BID is generally comparable to or, in some cases, superior to BAT. In this *post hoc* analysis, rates of fatal events, thrombosis, MACE, and nonmelanoma skin cancer were higher on ruxolitinib than on pacritinib. It is possible that differences in the kinome profiles between various JAK inhibitors [13] result in variability in the safety profile for individual drugs within this class, as each of the JAK inhibitors target various pathways beyond JAK/STAT. For example, unlike ruxolitinib, pacritinib does not inhibit JAK1, which is involved in the differentiation and activity of natural killer cells [14], and which may contribute to innate antitumoral and antiviral responses. Furthermore, pacritinib uniquely inhibits IRAK1, which modulates the toll‐like receptor pathway, although the clinical implications of IRAK1 inhibition remain under active investigation. It is also notable that rates of most events were higher in patients with platelet counts <50 × 10^9^/L regardless of treatment arm, likely reflecting more advanced disease biology for these severely cytopenic patients. This dataset supports the safe use of pacritinib 200 mg BID as a therapeutic option for patients with MF, including those with severe thrombocytopenia.

## AUTHOR CONTRIBUTIONS

NP, SB, KR‐T, and AY were involved in conception and design of the study. All authors participated in the analysis and interpretation of the data. SB drafted the manuscript. All authors critically revised the manuscript and provided final approval for submission and publication.

## CONFLICT OF INTEREST

Naveen Pemmaraju serves on the board of directors for Dan's House of Hope; consults for AbbVie, Aptitude Health, Astellas Pharma US Inc., Blueprint Medicines, Bristol‐Myers Squibb, Celgene, Cimeio Therapeutics AG, Clear View Healthcare Partners, CTI BioPharma, Dava Oncology, Immunogen, Incyte, Intellisphere LLC., Novartis, OncLive (owned by Intellisphere LLC), Patient Power, PharmaEssentia, Protagonist Therapeutics, Sanofi‐aventis, Stemline Therapeutics Inc., and Total CME; has served on scientific/advisory committees for Cancer.Net, CareDx, CTI BioPharma, EUSA Pharma Inc., Novartis, Pacylex, and PharmaEssentia; and reports speaker/preceptorship for AbbVie, Aplastic Anemia & MDS International Foundation, Curio Science LLC, Dava Oncology, Imedex, Magdalen Medical Publishing, Medscape, Neopharm, PeerView Institute for Medical Education, Physician Education Resource (PER), Physicians Education Resource (PER), Postgraduate Institute for Medicine, and Stemline Therapeutics Inc. Claire Harrison received honoraria from AbbVie, CTI BioPharma, Geron, Janssen, and Novartis; has served in consulting/advisory capacity for AOP, Celgene/ BMS, Constellation Pharmaceuticals, CTI BioPharma, Galecto, Geron, Gilead, Janssen, Keros, Promedior, Roche, Shire, Sierra Oncology, and Novartis; has served on a speakers bureau for AbbVie, BMS, CTI BioPharma, Geron, Sierra Oncology, and Novartis; and has received research funding from BMS, Constellation Pharmaceuticals, and Novartis. Vikas Gupta has consulted for AbbVie, Celgene/BMS, Constellation Pharmaceuticals, Novartis, Pfizer, and Sierra Oncology; he has received honoraria from Celgene/BMS, Constellation Pharmaceuticals, and Novartis; and has served in consulting/advisory capacity for AbbVie, Celgene/BMS, Pfizer, and Roche. Srdan Verstovsek has consulted for BMS, Constellation Pharmaceuticals, Incyte, Novartis, and Sierra Oncology; and has received researching funding from AstraZeneca, Blueprint Medicines, Celgene, CTI BioPharma, Genentech, Gilead, Incyte, Italfarmaco, Novartis, NS Pharma Inc., PharmaEssentia, Promedior, Protagonist Therapeutic, Roche, and Sierra Oncology. Bart Scott has consulted for Acceleron Pharma, Celgene, and Novartis; has served on speakers’ bureaus for Alexion Pharmaceuticals, Celgene, Jazz Pharmaceuticals, and Novartis; has received honoraria from BMS, Incyte, and Taiho Oncology, and reports his institution receiving research funding from Celgene. Stephen T. Oh has consulted for AbbVie, Blueprint Medicines, Celgene/BMS, Constellation Pharmaceuticals, CTI BioPharma, Disc Medicine, Geron, Incyte, and PharmaEssentia; and has received research funding from Actuate Therapeutics, Blueprint Medicines, Celgene/BMS, Constellation Pharmaceuticals, CTI BioPharma, Incyte, Kartos Therapeutics, Sierra Oncology, and Takeda. Francesca Palandri received honoraria and has served in consulting/advisory capacity for AOP, Celgene, CTI BioPharma, Novartis, and Sierra Oncology. Haifa Kathrin Al‐Ali has received grants from BMS, Deutsche Leukämie und Lymphom Stiftung, and East German Study Group for Hematology and Oncology; and has consulted for AbbVie, AOP, Blueprint Medicines, BMS, Novartis, Pfizer, and Takeda. Marta Sobas received honoraria and has served in consulting/advisory capacity for Celgene, CTI BioPharma, and Novartis. Mary Frances McMullin has served in consulting/advisory capacity for AbbVie, BMS, Incyte, Novartis, and Sierra Oncology; and has served on a speakers’ bureau for AbbVie, AOP, Incyte, Pfizer, and Novartis. Ruben Mesa has consulted for Constellation Pharmaceuticals, LaJolla Pharmacuetical, Novartis, and Sierra Oncology; has received research support from AbbVie, Celgene, Constellation Pharmaceuticals, CTI BioPharma, Genotech, Incyte, Promedior, and Samus; and has received a P30 grant (Mays Cancer Center P30 Cancer Center Support Grant) from National Cancer Institute (CA054174). Sarah Buckley is employed by, owns stock in, and has received travel funding from CTI BioPharma. Karisse Roman‐Torres is employed by and owns stock in CTI BioPharma. Alessandro Vannucchi has served in consulting/advisory capacity and has served on speakers’ bureaus for AbbVie, AOP, Blueprint Medicines, BMS, Incyte, and Novartis. Abdulraheem Yacoub has served in consulting/advisory capacity for AbbVie, Acceleron Pharma, Apellis, CTI BioPharma, Gilead, Incyte, Notable Labs., Novartis, Pfizer, PharmaEssentia, and Servier.

## ETHICS STATEMENT

PERSIST‐2 and PAC203 were approved by the institutional review boards at each institution and conducted in accordance of the principles outlined in the Declaration of Helsinki.

## Data Availability

Inquiries regarding the availability of data and clinical trial documentation will be considered on a case‐by‐case basis and should be directed to: Medinfo@ctibiopharma.com
